# Structural Features and In Vitro Antiviral Activities of Fungal Metabolites Sphaeropsidins A and B Against Bovine Coronavirus

**DOI:** 10.3390/ijms26157045

**Published:** 2025-07-22

**Authors:** Luca Del Sorbo, Maria Michela Salvatore, Clementina Acconcia, Rosa Giugliano, Giovanna Fusco, Massimiliano Galdiero, Violetta Iris Vasinioti, Maria Stella Lucente, Paolo Capozza, Annamaria Pratelli, Luigi Russo, Rosa Iacovino, Anna Andolfi, Filomena Fiorito

**Affiliations:** 1 Department of Veterinary Medicine and Animal Production, University of Naples Federico II, 80137 Naples, Italy; luca.delsorbo2@studenti.unina.it (L.D.S.); mariamichela.salvatore@unina.it (M.M.S.); rosa.giugliano@unicampania.it (R.G.); filomena.fiorito@unina.it (F.F.); 2 Department of Environmental, Biological and Pharmaceutical Sciences and Technologies, University of Campania Luigi Vanvitelli, 81100 Caserta, Italy; clementina.acconcia@unicampania.it (C.A.); luigi.russo2@unicampania.it (L.R.); 3 Istituto Zooprofilattico Sperimentale del Mezzogiorno, 80055 Portici, Italy; giovanna.fusco@izsmportici.it; 4 Department of Experimental Medicine, University of Campania Luigi Vanvitelli, 80138 Naples, Italy; massimiliano.galdiero@unicampania.it; 5 Department of Veterinary Medicine, University of Bari, 70010 Valenzano, Italy; violetta.vasinioti@uniba.it (V.I.V.); mariastella.lucente@uniba.it (M.S.L.); paolo.capozza@uniba.it (P.C.); annamaria.pratelli@uniba.it (A.P.); 6 Department of Chemical Science, University of Naples Federico II, 80126 Naples, Italy

**Keywords:** bovine coronavirus, antiviral secondary metabolites, aryl hydrocarbon receptor

## Abstract

The scientific community’s interest in natural compounds with antiviral properties has considerably increased after the emergence of the severe acute respiratory syndrome coronavirus (SARS-CoV-2), especially for their potential use in the treatment of the COVID-19 infection. From this perspective, bovine coronavirus (BCoV), member of the genus β-CoV, represents a valuable virus model to study human β-CoVs, bypassing the risks of handling highly pathogenic and contagious viruses. Pimarane diterpenes are a significant group of secondary metabolites produced by phytopathogenic fungi, including several *Diplodia* species. Among the members of this class of natural products, sphaeropsidin A (SphA) and its analog sphaeropsidin B (SphB) are well known for their bioactivities, such as antimicrobial, insecticidal, herbicidal, and anticancer. In this study, the antiviral effects of SphA and SphB were evaluated for the first time on bovine (MDBK) cells infected with BCoV. Our findings showed that both sphaeropsidins significantly increased cell viability in infected cells. These substances also caused substantial declines in the virus yield and in the levels of the viral spike S protein. Interestingly, during the treatment, a cellular defense mechanism was detected in the downregulation of the aryl hydrocarbon receptor (AhR) signaling, which is affected by BCoV infection. We also observed that the presence of SphA and SphB determined the deacidification of the lysosomal environment in infected cells, which may be related to their antiviral activities. In addition, in silico investigations have been performed to elucidate the molecular mechanism governing the recognition of bovine AhR (bAhR) by Sphs. Molecular docking studies revealed significant insights into the structural determinants driving the bAhR binding by the examined compounds. Hence, in vitro and in silico results demonstrated that SphA and SphB are promising drug candidates for the development of efficient therapies able to fight a β-CoV-like BCoV during infection.

## 1. Introduction

Fungi are a promising source of secondary metabolites belonging to different classes of natural products. These metabolites are low-molecular-weight compounds that play crucial roles for fungi, such as mediating communications, defense, and nutrient acquisition [1,2]. Thanks to their valuable bioactivities, including antibacterial, antifungal, antiviral, and cytotoxic, fungal metabolites find diverse applications and play an important role in the development of new drugs [3,4,5,6]. In fact, several pharmaceutical companies have actively investigated natural products and their derivatives as effective antimicrobial drugs potentially able to counteract viral and bacterial infections [7,8,9,10].

The past few years have seen an explosion in the scientific community’s interest in the antiviral properties of fungal metabolites [11,12,13]. In fact, researchers are engaged in intense investigation for finding novel antivirals to fight emerging pathogenic coronaviruses (CoVs). In particular, the recent coronavirus disease 2019 (COVID-19) pandemic, caused by the severe acute respiratory syndrome coronavirus 2 (SARS-CoV-2), has drawn attention to CoVs’ ability to mutate into new and more dangerous variants [14,15,16].

From the perspective of identifying natural antiviral drugs, the use of animal CoVs, represents a valid alternative model to carry out preliminary studies on the efficacy of drugs, avoiding the risks deriving from the use of a highly pathogenic and contagious virus for the first screening phase. In this respect, studies on the antiviral properties of fungal metabolites have been conducted against animal CoVs, and the promising results obtained pave the way for further investigations [17,18,19,20].

Classical antiviral drugs mainly play a role as a peculiar target acting on viral replication, including SARS-CoV-2 [21,22,23,24,25]. Due to the considerable ability of viruses, especially RNA viruses, to provoke the recombination of their genetic material, in order to obtain a broad-spectrum application, a durable efficacy, as well as a reduced attitude for promoting the acquisition of drug resistance, host-targeted compounds have been recently developed [24,25]. For the aryl hydrocarbon receptor (AhR), a transcription factor that is activated by endogenous and exogenous compounds (e.g., bilirubin, biliverdin, metabolites from the essential amino acid L-tryptophan, environmental pollutants (like dioxins), some drugs, and microbial metabolites), mainly known for its role in detoxifying environmental pollutants, its functions have recently been established in other physiological processes, including the cellular cycle and differentiation, lipid and carbohydrate metabolisms, as well as the control of the immune system in viral infections [26,27]. Particularly, during RNA viral infections caused by the human immunodeficiency virus, influenza A virus, Zika virus, and CoVs, AhR activation, by modifying cytokine levels, is able to induce changes in hosts’ immune responses, supporting a mechanism for the evasion of the host’s antiviral immune response and, consequently, promoting viral replication [26,27]. About CoVs, AhR is upregulated by infections due to both alpha-CoVs, including HCoV-229E, canine coronavirus (CCoV), porcine epidemic diarrhea virus (PEDV), as well as feline coronavirus (FCoV), and beta-CoVs, like mouse hepatitis virus (MHV), HCoV-OC43, MERS-CoV, SARS-CoV-1, SARS-CoV-2, and BCoV [28,29,30,31,32,33,34,35,36]. Notably, AhR is stimulated by SARS-CoV-2 infection, while when AhR is pharmacologically inhibited, the suppression of SARS-CoV-2 replication has been revealed in different cell lines [29,30,31]. In addition, our previous studies have revealed the involvement of AhR in the antiviral actions of some fungal secondary metabolites, including funicone-like compounds (3-*O*-methylfunicone, vermistatin, and penisimplicissin) produced by *Talaromyces pinophilus* [17,18,19] and 6-pentyl-α-pyrone obtained from *Trichoderma atroviridae* [20]. Hence, AhR might be further characterized, through in vitro and in vivo studies, as an attractive target for antivirals useful for improving the host’s response to CoV infections. Thus, to counteract CoV infections, we have proposed to indagate this emergent mechanism of action, different from those of most classical antiviral drugs, which generally act on the replication mechanism of coronaviruses or in the assembly phase [21,22,23,24,25].

**Figure 1:**
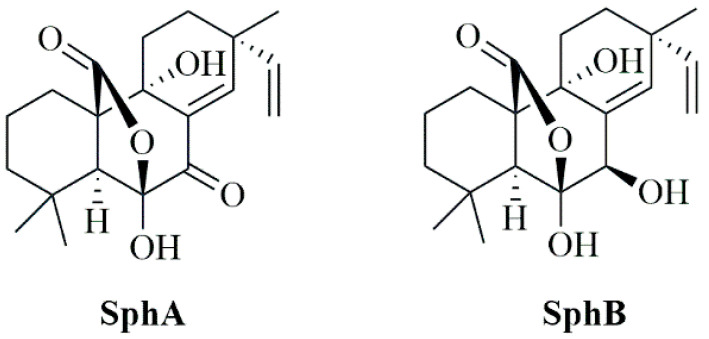
Chemical structures of SphA and SphB.

In the present work, we explored the potential antiviral effects of sphaeropsidin A (SphA) and its analog sphaeropsidin B (Sph B) (Figure 1), two secondary metabolites produced by the fungus *Diplodia corticola*. SphA and SphB are well-known pimarane diterpenes frequently isolated from fungi of the genus *Diplodia* [37]. Due to their effective antimicrobial, insecticidal, herbicidal, and anticancer activities, these metabolites are considered as promising substances for use in both agriculture and medicine [38,39,40].

The experimental design of this study started with the determination of the non-toxic in vitro doses of SphA and SphB in Madin–Darby Bovine Kidney (MDBK) cells. Subsequently, the antiviral activities of these metabolites against BCoV were evaluated, revealing that SphB was active at 1 µM, while SphA was active at 2.5 µM, by investigating the expression of AhR during BCoV infection in the presence of these fungal metabolites.

Our strategy allowed the support of favorable results obtained from the in vitro investigations with computational studies, which provided significant insights into the molecular determinants governing the recognition of the bovine AhR by SphA and SphB. So, the antiviral activities of SphA and SphB are reported for the first time.

## 2. Results

### 2.1. SphA and SphB Increased Cell Viability During BCoV Infection

**Figure 2:**
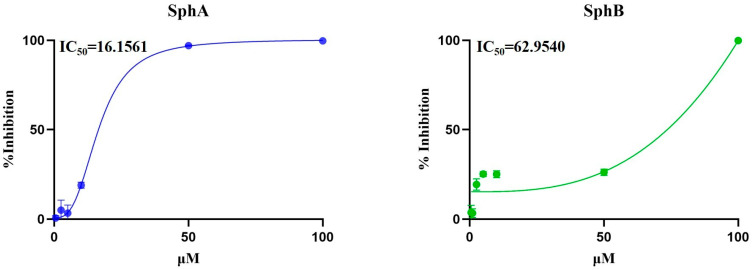
Determination of the IC_50_ values of SphA and SphB at different concentrations (0.5, 1, 2.5, 5, 10, 50, and 100 µM) and the development of dose–response curves for MDBK cells after 120 h of treatment. Dose–response curves of MDBK cells treated with SphA and SphB at different concentrations, and the cell viability was assessed using TB staining and measured using an automated cell counter. The number of living cells over the total cell number was determined, and the inhibition percentage was calculated and reported as the mean of triplicates ± S.D. The percentage of inhibition was calculated against the DMSO control and analyzed in GraphPad Prism 10.5.0 via non-linear regression with a dose–response inhibition model (four parameters). Points represent the mean of triplicates ± S.D.s (some S.D. values were too low and were omitted).

The effects of different concentrations of SphA and SphB (0.5, 1, 2.5, 5, 10, 50, and 100 µM) were assessed on MDBK cells after 120 h of treatment. The cytotoxic concentrations of these compounds that reduced cell viability by 50% (IC_50_) in MDBK cells allowed the obtention of dose–response curves (Figure 2). Cell viability (as a percentage of the control) was calculated at 120 h of treatment, with IC_50_ values of 16.1561 μM for SphA and 62.9540 μM for SphB (Figure 2).

Next, MDBK monolayers were infected with BCoV at a multiplicity of infection (MOI) of 0.05 and treated or not with SphA and SphB at different concentrations (0.5, 1, 2.5, 5, 10, 50, and 100 µM). After 120 h of infection, the values of the effective 50% (EC_50_) concentrations for SphA and SphB were calculated, and EC_50_ = 12.470 for BCoV + SphA and EC_50_ = 59.363 for BCoV + SphB were obtained.

**Figure 3:**
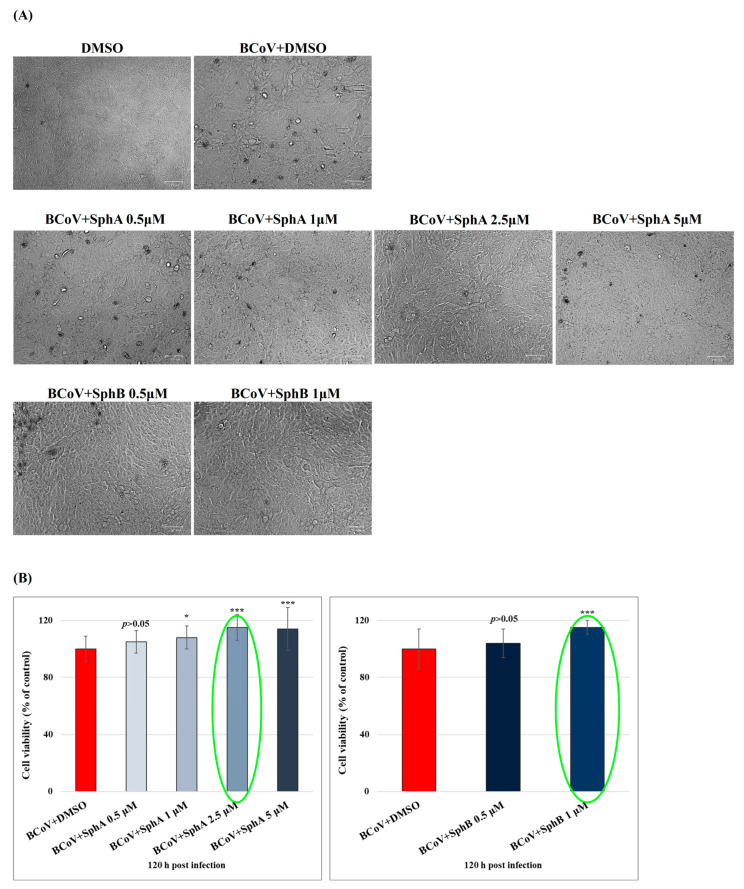
SphA and SphB enhanced cell viability during BCoV infection. (**A**) MDBK cells treated or not with SphA at 0.5, 1, 2.5, and 5 µM (and infected with BCoV) and with SphB at 0.5 and 1 µM (and infected with BCoV). At 120 h p.i., the cells were stained with TB and observed under a light microscope. Scale bar represents 50 µm. (**B**) Bar graphs showing dose–response effects of the indicated concentrations of SphA and SphB on MDBK cells infected with BCoV. The selected concentrations for SphA and SphB are indicated with the green circle. After 120 h of infection, the cell viability was assayed using TB staining and scored using an automated cell counter. The number of living cells over the total cell number was determined as a percentage and the results were reported as the mean of triplicates ± S.D.s. Points represent one-way ANOVA with Tukey’s post hoc test. Significant differences between the BCoV + DMSO- and BCoV + SphA-treated cells as well as between the BCoV + DMSO- and BCoV + SphB-treated cells are highlighted; probabilities: ***, *p* < 0.001 and *, *p* < 0.05.

In Figure 3, we displayed and analyzed the effects of the selected concentrations of SphA (0.5, 1, 2.5, and 5 µM) and SphB (0.5 and 1 µM) added on BCoV-infected cells (MOI = 0.05). After 120 h of infection, SphA at 2.5 µM and SphB at 1 µM induced significant (*p* < 0.001) enhancements in the MDBK cell viability of infected cells (Figure 3).

### 2.2. SphA and SphB Reduced Signs of the Cell Death Morphology During BCoV Infection in MDBK Cells

**Figure 4:**
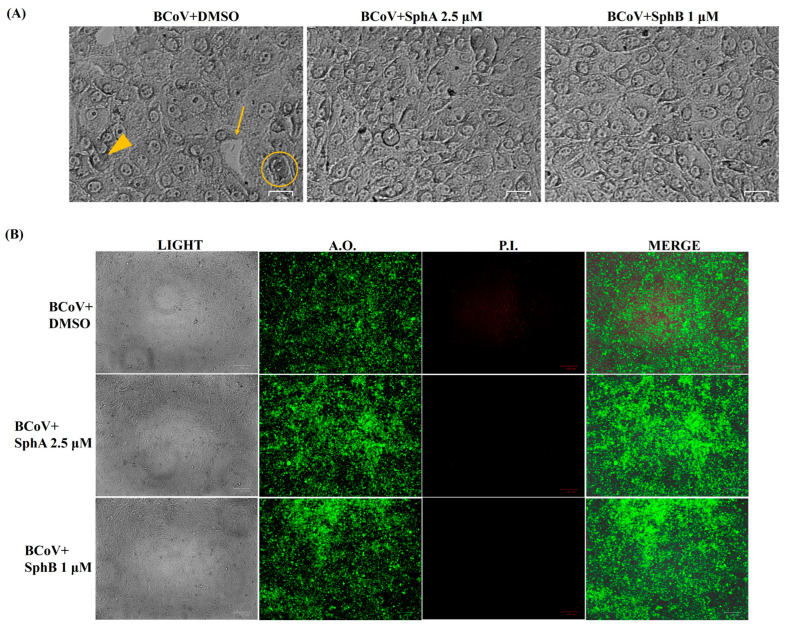
SphA and SphB decreased the morphological signs of cell death during BCoV infection in MDBK cells. Cells treated or not with SphA (2.5 µM) and SphB (1 µM) were infected with BCoV. (**A**) At 96 h p.i., the cells were stained with Giemsa’s solution and observed under a light microscope. Signs of morphological cell death, like cellular shrinkage (arrowhead), pyknosis (circle), and intercellular spaces due to the detachment of cells from the culture plate (slim arrow), were reduced in SphA- and SphB-treated groups compared to the controls. (**B**) In acridine orange/propidium iodide (AO/PI) panels, PI fluorescent cells, showing dead and dying cells, were mostly detected in BCoV-infected cells compared to both the SphA- and SphB-treated BCoV-infected cells. Scale bars represent 25 µm and 100 µm. The results of one experiment, representative of three independent experiments, were reported.

By employing Giemsa staining, it was possible to detect signs of the cell death morphology. In detail, in infected and dimethyl sulfoxide (DMSO)-treated groups, cellular shrinkage (Figure 4, arrowhead) and pyknosis (Figure 4, circle) were detected. These cell death signs were diminished in BCoV-infected cells in the SphA- and SphB-treated groups compared to the controls (Figure 4). Moreover, the enhancement of intercellular spaces produced by cell detachment from the culture plate was mainly observed in untreated BCoV-infected cells (slim arrow) compared to the control groups (Figure 4). In the acridine orange/propidium iodide (AO/PI) panels, PI fluorescent cells, indicating dead and dying cells, were mostly detected in BCoV-infected cells compared to the Sph-treated BCoV-infected cells (Figure 4).

These findings showed that SphA and SphB greatly protected bovine cells during BCoV infection.

### 2.3. SphA and SphB Induced Reductions in the Virus Yield During BCoV Infection

**Figure 5:**
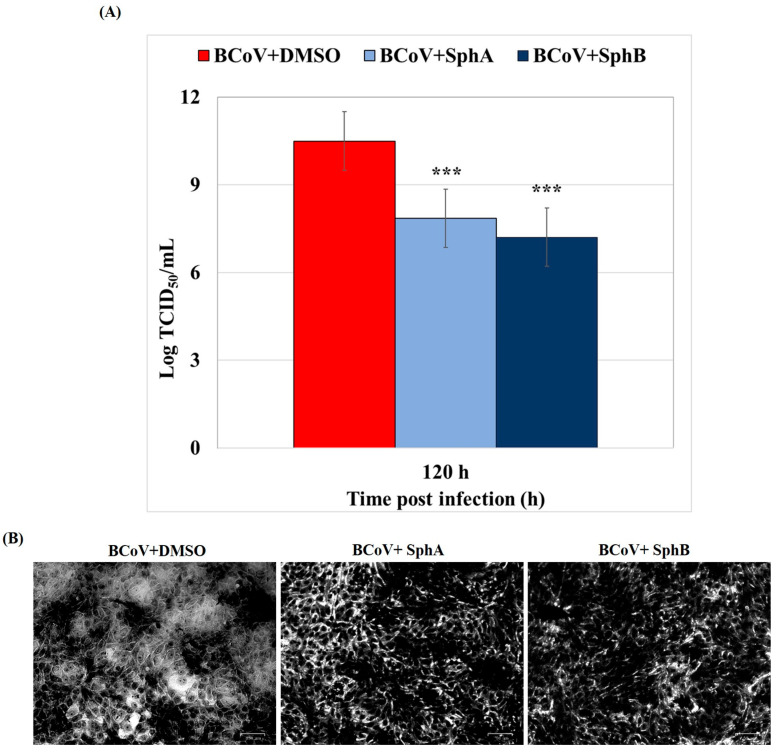
SphA and SphB induced reductions in the virus yield during BCoV infection in MDBK cells. MDBK cells were infected with BCoV and treated or not with SphA (2.5 µM) and SphB (1 µM) for 120 h p.i. (**A**) The virus yield was assessed using the TCID_50_ method and reported as Log TCID_50_/mL. Student’s t-test was performed. Significant differences between the BCoV-infected group and both the SphA- and SphB-infected cells are marked; probability: ***, *p* < 0.001. (**B**) CPE by crystal violet staining was assayed using a ZOE Cell Imager. Scale bar represents 50 µm. The results of one experiment, representative of three independent experiments, were reported.

Following BCoV infection in MDBK cells, SphA (2.5 µM) and SphB (1 µM) affected the virus’s production, as the BCoV virus titers (expressed as logarithms) at 120 h p.i. resulted in significantly reduced (*p* < 0.001) in infected cells treated with both fungal metabolites (Figure 5A) compared to the BCoV + DMSO control cells.

Following crystal violet staining, the microscopic analysis of the infected groups revealed an increase in the cytopathic effect (CPE) when untreated cells were compared to the SphA- and SphB-treated groups (Figure 5B).

Taken together, our findings demonstrated that both fungal metabolites, SphA and SphB, provoked significant reductions in the BCoV yield during infection in MDBK cells. It must be noted that SphB was already active at a concentration of 1 µM, while SphA was active at 2.5 µM.

### 2.4. SphA and SphB Reduced NP Gene Expression During BCoV Infection in MDBK Cells

**Figure 6:**
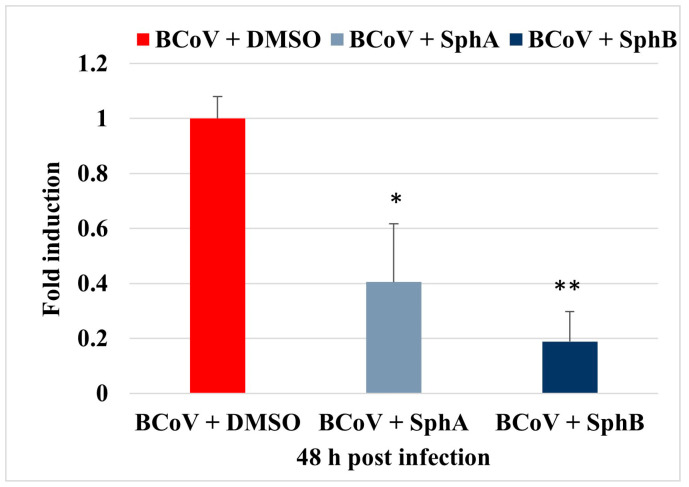
SphA and SphB provoked reductions in NP gene expression during BCoV infection in MDBK cells. The qPCR analysis of the NP gene expression in BCoV after treatments with SphA and SphB shows that both metabolites significantly reduced the gene expression compared to that of the untreated BCoV. Error bars represent the standard deviations of the measurements. One-way ANOVA with Tukey’s post hoc test was performed. Significant differences in the NP gene expressions between the BCoV + DMSO and both the BCoV + SphA and BCoV + SphB groups are indicated; probabilities: **, *p* < 0.01 and *, *p* < 0.05. The results of one experiment, representative of three independent experiments, were reported.

The real-time results indicated that treatments with SphA and SphB significantly reduced the gene expression of the viral nucleoprotein (NP) compared to the untreated BCoV. Both metabolites demonstrated significant viral inhibition, with SphB exhibiting a more pronounced effect compared to that of SphA (Figure 6).

### 2.5. SphA and SphB Caused Reductions in Cellular AhR and Viral S Protein Expressions During BCoV Infection

**Figure 7:**
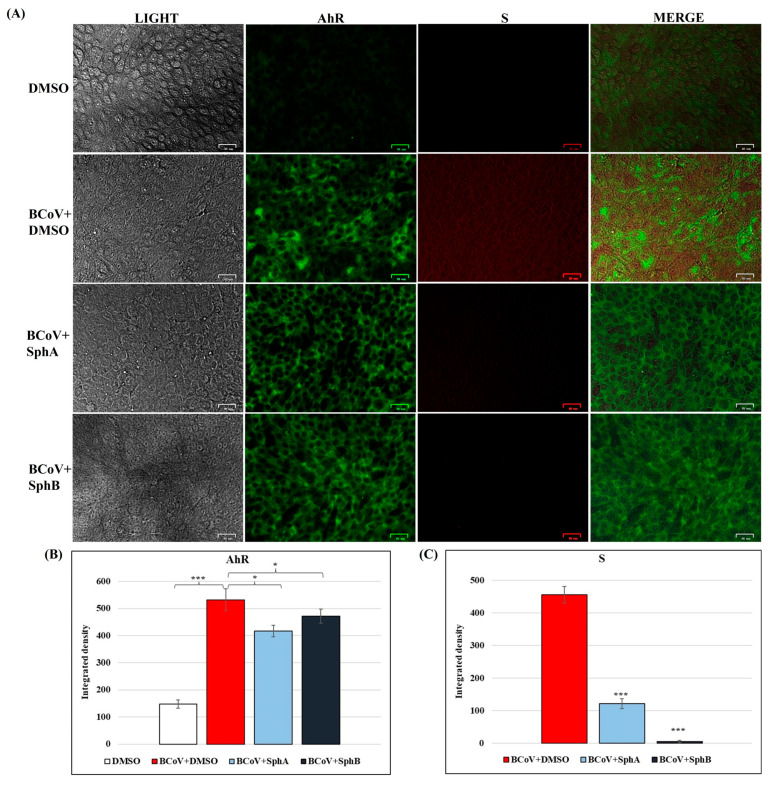
SphA and SphB determined the downregulation of the expressions of AhR and S proteins during BCoV infection in MDBK cells. MDBK cells were infected with BCoV at an MOI of 0.05 and treated or not with SphA (2.5 µM) and SphB (1 µM) for 48 h. (**A**) In the SphA- and SphB-treated and untreated BCoV-infected groups, the levels of the AhR and S proteins were tested using immunofluorescence staining. Scale bar represents 25 µm. (**B**,**C**) Bars show the mean ratios generated from the integrated densities (the product of the area and the mean fluorescence intensity) of the AhR and S protein expressions during BCoV infection. One-way ANOVA with Tukey’s post hoc test was performed. Significant differences between the control (DMSO-treated) and BCoV-infected cells, as well as between the BCoV-infected cells and both the SphA-treated infected cells and SphB-treated infected cells for the AhR and S protein levels are indicated; probabilities: ***, *p* < 0.001 and *, *p* < 0.05. The integrated densities were assessed using ImageJ (National Institutes of Health) software (Java 1.8.0_345). Error bars represent the standard deviations of the measurements. The results of one experiment, representative of three independent experiments, were reported.

As previously demonstrated [36], BCoV infection is responsible of the activation of AhR (Figure 7A). However, during BCoV infection, immunofluorescence staining revealed reductions in the expressions of AhR when MDBK cells were treated with SphA (2.5 µM) and SphB (1 µM) (Figure 7A). As a consequence, the integrated densities of the AhR immunofluorescence images turned out to be significantly (*p* < 0.05) decreased, as seen in Figure 7B.

Interestingly, the S protein expression was also analyzed during BCoV infection in MDBK cells (Figure 7). The level of the S protein was strongly reduced in infected groups treated with both SphA and SphB compared to that in the untreated infected cells (Figure 7A). These results were supported by the integrated density fluorescence quantification (Figure 7B), which was significantly (*p* < 0.001) decreased.

### 2.6. SphA and SphB Caused Reductions in CYP1A1 Protein Levels During BCoV Infection

**Figure 8:**
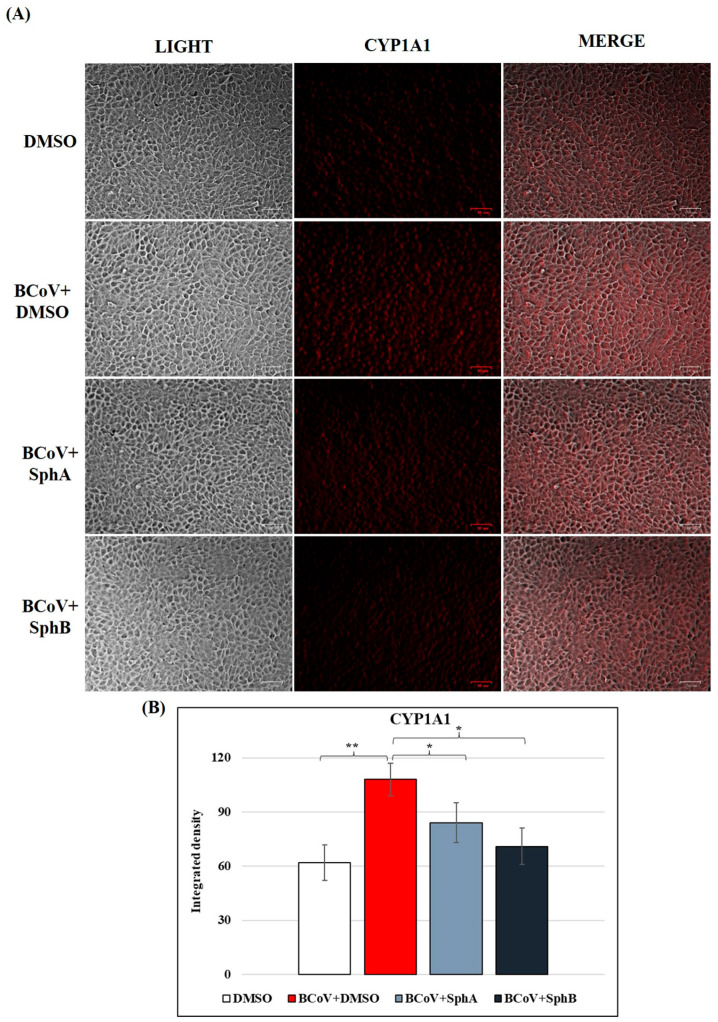
SphA and SphB downregulated the expression of CYP1A1 (AhR signaling) during BCoV infection in MDBK cells. MDBK cells were infected with BCoV at an MOI of 0.05 and treated with SphA and SphB for 48 h. Then, immunofluorescence staining with antibodies recognizing (**A**) CYP1A1 was carried out. Scale bar represents 50 µm. (**B**) Bars show the mean ratios generated from the integrated densities (the product of the area and the mean fluorescence intensity) of the CYP1A1 expressions during BCoV infection in the presence of SphA and SphB. One-way ANOVA with Tukey’s post hoc test was performed. Significant differences between the DMSO and BCoV-infected cells, as well as between the BCoV-infected cells and both the SphA- and SphB-treated infected cells for the CYP1A1 protein are indicated; probabilities: *, *p* < 0.05 and **, *p* < 0.01. The integrated densities were measured using ImageJ. Error bars represent the standard deviations of the measurements. The results of one experiment, representative of three independent experiments, were reported.

To measure the effects of SphA and SphB on the expression of AhR signaling, the level of cytochrome CYP1A1 was analyzed during BCoV infection in MDBK cells. The upregulation of CYP1A1 due to BCoV infection was significantly reduced by treatments with SphA and SphB (Figure 8A). The integrated density of immunofluorescence images further demonstrated the significant reduction in CYP1A1 due to treatments with both SphA and SphB (*p* < 0.05), as reported in Figure 8B.

### 2.7. SphA and SphB Deacidified Lysosomes During BCoV Infection in MDBK Cells

**Figure 9:**
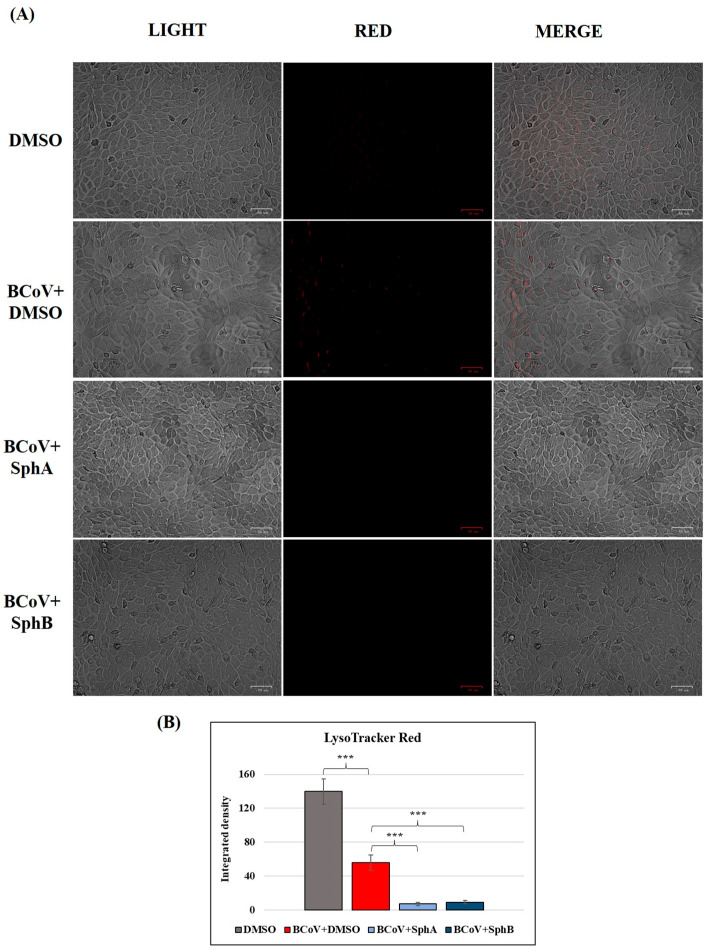
SphA and SphB deacidified lysosomes during BCoV infection in MDBK cells. MDBK cells were infected with BCoV at an MOI of 0.05 and treated or not with SphA (2.5 µM) and SphB (1 µM) for 72 h. (**A**) LysoRed staining of the DMSO control group compared to those of the untreated BCoV-infected cells and the SphA- and SphB-treated BCoV-infected groups. Scale bar represents 50 µm. (**B**) Bars indicate the mean ratios obtained by the integrated densities of the LysoTracker obtained using ImageJ. One-way ANOVA with Tukey’s post hoc test was performed. Significant differences between the DMSO and BCoV-infected cells, as well as between the BCoV-infected cells and both the SphA- and SphB-treated infected cells are indicated; probability: ***, *p* < 0.001. The results of one experiment, representative of three independent experiments, were reported.

The acidic environment of the cellular organelles’ lysosomes was analyzed during infection, using LysoRed staining, a marker for the identification lysosomes in live cells. In the DMSO control cells, we detected an acidic environment that was deacidified by BCoV infection (Figure 9A,B). The treatments of the BCoV-infected cells with SphA (2.5 µM) and SphB (1 µM) caused further deacidification of the lysosomes (Figure 9A,B).

### 2.8. Molecular Docking Analysis of bAhR in Complexes with SphA and SphB

**Figure 10:**
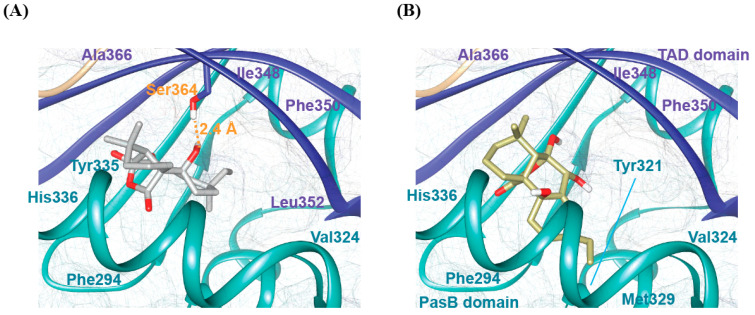
Docking-based structural analysis of SphA and SphB binding to both PasB (cyan) and TAD (blue) domains of bAhR receptor. (**A**) Structural model of SphA within the binding pocket of the bovine aryl hydrocarbon receptor (bAhR). The residues involved in the interactions are hydrogen bonding (Ser364) and hydrophobic interactions (Phe294, Tyr335, His336, Val324, Phe350, Leu352, Ile348, and Ala366), as shown in the figure. (**B**) Predicted structural model of SphB within the same ligand-binding pocket of the bAhR. In this case, the interactions are stabilized by hydrophobic contacts involving residues such as Phe294, Tyr321, His336, Val324, Met329, Phe350, Ile348, and Ala366.

To elucidate the molecular mechanisms by which SphA and SphB interact with the bovine AhR (bAhR) receptor (Figure S1), we performed, as reported in the materials and methods section, a series of molecular docking studies. After that, we validated the obtained structural models by evaluating the stability of each complex through the estimation of the binding energy, using the web server PRODIGY. We obtained identical binding energies of −5.47 kcal/mol for both SphA/bAhR and SphB/bAhR, indicating that both complexes are characterized by reasonable stability. The representative three-dimensional docking models showed that the two ligands are accommodated in a shallow hydrophobic cleft located between the PasB and TAD domains, indicating that the recognition mechanism, in both cases, is mainly driven by hydrophobic interactions. However, an accurate analysis of the docking results revealed that SphA and SphB are characterized by slightly different binding modes in terms of their relative orientations with respect to the bAhR domains. In detail, the docking model of the SphA/bAhR complex demonstrated that the ligand is predominantly stabilized by a network of hydrophobic contacts with key residues, including Phe294, Val324, Tyr335, His336, Ile348, Phe350, Leu352, and Ala366 (Figure 10A; Table S1). Additionally, the SphA/bAhR complex is further reinforced by a specific hydrogen bond between the ligand and Ser364. SphB, although it occupies the same binding pocket as that occupied by SphA, exhibits a slightly different interaction profile. In particular, the binding of SphB to the receptor is totally mediated by hydrophobic interactions involving a large number of residues surrounding the binding pocket (e.g., Phe294, Tyr321, Val324, Met329, His336, Ile348, Phe350, and Ala366) (Figure 10B; Table S2). Overall, our docking studies provided compelling evidence that both ligands recognize the same hydrophobic pocket of the bAhR receptor with slightly different binding mechanisms (Figure 10A,B). Of note, SphA and SphB show interaction profiles similar to that previously observed for the CH223191 inhibitor [36], highlighting the presence in the receptor of a conserved binding site composed of key residues playing a crucial role in the ligands’ recognition mechanism.

## 3. Discussion

In a framework characterized by extensive research aimed at reducing the diffusion of SARS-CoV-2 infections [21,22,23,41], this work focused on identifying novel natural compounds with antiviral properties. To achieve this goal, a translational approach has been developed using BCoV, a β-CoV like SARS-CoV-2, and MDBK host cells. In particular, the promising bioactive compounds belonging to pimarane diterpenes, i.e., SphA and SphB, have been selected to investigate their potential antiviral properties.

Non-toxic doses of these compounds (SphA at 2.5 µM and SphB at 1 µM) were determined and selected for further investigations. During BCoV infection, SphA and SphB induced reductions in the virus titer and in the expressions of the viral S protein, as well as in NP gene expressions. Under the same experimental conditions, increased cell viability and reduced signs of cell death were also observed in bovine cells (MDBK). These results were observed in the presence of a significant reduction in the expression of the AhR. Interestingly, following BCoV infection in the same in vitro system (MDBK), similar results were obtained with the addition of CH223191, a well-known inhibitor of AhR [36]. AhR is a transcription factor, which, after being regulated by endogenous and exogenous compounds, acts as a modulator of natural host immune responses, modifying the values of cytokines [26]. The role of the AhR in host cell–pathogen interactions is extensively recognized [26,42]. Regarding CoVs, including both α- and β-CoVs, remarkable upregulations of the AhR were recently revealed during hosts’ responses to infections with mouse hepatitis virus (MHV), Middle East respiratory syndrome coronavirus (MERS-CoV), human coronaviruses (HCoV229E, SARS-CoV-1, and SARS-CoV-2), canine coronavirus (CCoV), porcine epidemic diarrhea virus (PEDV), and feline coronavirus (FCoV) [28,29,30,31,32,33,34,35].

In our study, during BCoV infection, the presence of SphA and SphB also provoked the downregulation of CYP1A1, a downstream target protein in the AhR pathway, which turned out to be increased in the untreated infected groups. Hence, we observed a modulation comparable to those of other compounds that were tested to inhibit CoV infections [29,30,31,35].

Beta-CoVs, including MHV and SARS-CoV-2, use endocytosis to enter host cells [43,44]. On the topic of SARS-CoV-2 infection, lysosomes and lysosomotropic treatments have been extensively reviewed [45,46,47]. Indeed, the deacidification of environmental cells was detected after CoV infection, a phenomenon also observed during in vitro infection with other CoVs, such as CCoV [19] and FCoV [35]. Interestingly, in this study, the deacidification of lysosomes was detected after 72 h of BCoV infection, and treatments with SphA and SphB not only protected the bovine cells infected by BCoV but further alkalinized the lysosomes of the infected cells. Similar results were obtained using small molecules that alkalinized lysosomes during SARS-CoV-2 infection in Vero E6 cells [48], as well as using the funicone-like compounds vermistatin and penisimplicissin following CCoV infection in a canine fibrosarcoma cell line (A72) [19]. Nevertheless, an acidic environment seems to be unrequired for BCoV infection, and BCoV-induced membrane fusion appears to be independent of low pH values [49], so this phenomenon requires more detailed studies.

In this study, the potential antiviral activities of natural compounds, like SphA and SphB, were reported in MDBK cells, highlighting that SphB was active at 1 µM, while SphA was active at 2.5 µM. The molecular docking studies provided significant insights into the molecular determinants governing the recognition of the bAhR receptor by SphA and SphB. Our results revealed that both ligands bind within the same hydrophobic pocket of the bAhR receptor, situated between the PasB and TAD domains, but they exhibited distinct interaction profiles. In particular, the formation of the SphA/bAhR complex is governed by a network of hydrophobic contacts, and it is further stabilized by a specific hydrogen bond between the ligand and Ser364, whereas SphB recognizes the receptor through a large number of hydrophobic interactions. Overall, the molecular docking results indicate that the two ligands recognize the same hydrophobic pocket of the bAhR receptor with slightly different binding mechanisms. Moreover, the comparison of the structural models obtained for SphA and SphB in complexes with the bAhR with that previously calculated for the inhibitor CH223191 highlights that the receptor is characterized by the presence of a conserved binding region composed of key residues playing a crucial role in the ligands’ recognition mechanism. These findings offer valuable insights into the molecular determinants of the ligand recognition and pave the way for the design of novel receptor modulators.

Moreover, these results were supported by computational studies showing the efficacy of SphA against dengue virus, another RNA virus [50]. The molecular docking analysis carried out to assay the binding affinities of natural compounds, including the pimarane diterpene SphA, to dengue viral proteins (the envelope, NS1, NS3, and NS5) showed good binding affinities [50], indicating promising antiviral properties.

In conclusion, our results present SphA and SphB as potential drugs suitable against BCoV infection and pave the way for the application of these fungal compounds and/or their derivatives as antivirals in the first phases of the treatment of other β-CoVs. However, further investigations are needed to test the activities of these compounds on additional cell lines and BCoV strains, as well as on other members of the AhR pathway.

## 4. Materials and Methods

### 4.1. Production, Isolation, and Identification of Sphaeropsidin A and Sphaeropsidin B

Sphaeropsidins A and B, employed in this study, were isolated from cultures of the fungus *Diplodia corticola* (MAEC10) recovered from *Quercus suber* in Algeria. A liquid culture of *D. corticola* was prepared as previously reported by Salvatore et al. [51]. Briefly, 250 mL of Czapek-Dox broth (Oxoid, Thermo Scientific, Waltham, MA, USA) amended with 2% cornmeal was inoculated with mycelial plugs from actively growing cultures and inoculated in 500 mL Erlenmeyer flasks. The culture was incubated in darkness on a stationary phase at 25 °C. After 30 days, the culture filtrate was obtained by filtrating the liquid culture through Whatman No. 5 filter paper. The culture filtrate was extracted three times with ethyl acetate (EtOAc) at the native pH (6). The organic phase was dried with anhydrous Na_2_SO_4_ and evaporated under reduced pressure, yielding a crude extract as a brownish solid residue. The crude extract was submitted to some purification steps by column chromatography and thin-layer chromatography on silica gel, eluted with diverse polar solvents.

Sphaeropsidin A (a white solid) and sphaeropsidin B (a white solid) were identified by comparing NMR data with previously reported data [51]. ^1^H NMR spectra (Figures S2 and S3) were recorded on a Bruker AMX instrument at 400 MHz in CDCl_3_. The same solvents were used as internal standards.

### 4.2. Cell Cultures and Virus Infection

MDBK (ATCC CCL 22) cell cultures were prepared in Dulbecco’s modified Eagle’s minimal essential medium (DMEM) with a supplement of 10% fetal bovine serum (FBS) [52]. BCoV, strain 282/23, collected by the Sector of Infectious Diseases in the Department of Veterinary Medicine (at the University of Bari Aldo Moro in Valenzano, Italy) [36], was cultured and titrated in MDBK cells.

SphA and SphB were dissolved in DMSO (Sigma-Aldrich, St. Louis, MI, USA) to have a dose of 5000 μM (stock solution). Then, working solutions of 0.5, 1, 2.5, 5, 10, 50, and 100 µM of SphA and SphB in DMEM were prepared in order to obtain the final concentrations to be added to the cell cultures. DMSO in DMEM (0.1% *v/v*) was used as a vehicle control.

MDBK cells were either infected or not infected with a tissue culture of an infectious dose 50 value (TCID_50_) of 1 × 10^8.70^/mL of BCoV strain 282/23, at an MOI of 0.05 or 0.5, and treated with DMEM supplemented with 10% FBS containing different concentrations of both SphA and SphB (0.5, 1, 2.5, 5, 10, 50, and 100 µM) to have six groups: (a) untreated uninfected cells, (b) untreated infected cells, (c) SphA-treated uninfected cells, (d) SphB-treated uninfected cells, (e) SphA-treated infected cells, and (f) SphB-treated infected cells. After 1 h of BCoV adsorption at 37 °C, the cells were incubated and processed at 48, 72, 96, and 120 h post infection (p.i.). BCoV was in the culture medium for the entire experiment.

### 4.3. Cell Viability

To establish the IC_50_ values of the SphA and SphB at various concentrations and elaborate the dose–response curve for MDBK cells, a Trypan Blue (TB) (Sigma-Aldrich, St. Louis, MI, USA) exclusion test was assessed as previously reported [53]. In particular, MDBK cell monolayers were treated with both SphA and SphB at different concentrations (0.5, 1, 2.5, 5, 10, 50, and 100 µM) and incubated for 120 h. After this, the cells were trypsinized, mixed with TB, and then counted using a TC20 automated cell counter (Bio-Rad Laboratories, Hercules, CA, USA). The number of living cells over the total cell number was determined, and the inhibition percentage was calculated and reported as the mean of triplicates ± S.D. The inhibition percentage was calculated against the DMSO control and analyzed in GraphPad Prism via non-linear regression with a dose–response inhibition model (four parameters) (Figure 2).

Cell viability (IC_50_) calculations for the MDBK cells, as well as the concentration of the effective 50% (EC50) values for SphA and SphB, which correspond to a 50% inhibition of the viral replication, were evaluated using the IC_50_ Calculator|AAT Bioquest (https://www.aatbio.com/tools/ic50-calculator (accessed on 5 March 2025)).

Then, the cells were infected or not infected with BCoV at an MOI of 0.05 and treated with the selected concentrations of SphA (0.5, 1, 2.5, and 5 µM) or SphB (0.5 and 1 µM). At 120 h p.i., the cells were trypsinized, mixed with TB, and then counted using a TC20 automated cell counter (Bio-Rad Laboratories, Hercules, CA, USA). The number of living cells over the total cell number was determined as a percentage, and the results were reported as the mean ± S.D. of three independent experiments.

### 4.4. Examination of the Cell Morphology

Cell monolayers, treated or not with SphA at a concentration of 2.5 μM and SphB at a concentration of 1 μM, were infected or not with BCoV (MOI = 0.05) and incubated for 96 h. After that, the cells were washed with PBS and stained with Giemsa’s solution and AO/PI [20,54,55]. The morphological features of cell death were then detected using microscopy [56,57,58].

Particularly, Giemsa staining was performed on fixed (95% ethanol), drained, and dried cells, staining with a 5% Giemsa solution (Merck, Darmstadt, Germany) for 30 min. The cells were subsequently rinsed (with tap water and H_2_O) and monitored using light microscopy (ZOE Cell Imager, Bio-Rad Laboratories, Hercules, CA, USA).

AO/PI staining was performed using fluorescence microscopy (ZOE Cell Imager, Bio-Rad Laboratories, Hercules, CA, USA) to recognize viable and dead cells [54]. Green fluorescence is the result of the binding of the AO (membrane permeability) to nucleic acids; a bright red fluorescent complex is obtained by the crossing of the PI through dead and dying cells, where it intercalates nucleic acids, while intact cell membranes become impermeable to the PI.

### 4.5. Immunofluorescence Staining

MDBK cells, treated or not with SphA (2.5 μM) and SphB (1 μM) were infected or not with BCoV, at an MOI of 0.5. Immunofluorescence staining was assessed at 48 h p.i. [18,59] using the following antibodies and antisera diluted in 5% bovine serum albumin, 1x Tris-buffered saline, and 0.1% Tween^®^ 20 detergent: (a) anti-aryl hydrocarbon receptor (AhR) (Sigma-Aldrich, St. Louis, MI, USA) (1:250), (b) mouse anti-bovine coronavirus spike antibody (5A4) (MAB12430, The Native Antigen Company, Oxford, UK) (1:400), (c) mouse monoclonal anti-CYP1A1 (A-9) (sc-393979, Santa Cruz Biotechnology, Inc., Dallas, TX, USA), (d) goat anti-rabbit Texas Red (Thermo Fisher Scientific, Waltham, MA, USA) (1:500), and (e) goat anti-mouse Alexa Fluor 488 (Thermo Fisher Scientific, Waltham, MA, USA) (1:500). The fluorescence signals from microscopy images, assessed using a ZOE Fluorescent Cell Imager (Bio-Rad Laboratories), were determined using ImageJ (National Institutes of Health, Bethesda, MD, USA) software.

### 4.6. Virus Production

MDBK monolayers, in a 24-well plate, were either infected or not with BCoV at an MOI of 0.5, treated or not with SphA (2.5 μM) and SphB (1 μM), and incubated at 37 °C for 120 h of infection. At 120 h p.i., after three freezing and thawing cycles, the cells were aliquoted and stored at −80 °C, and virus titration was assessed using the TCID_50_ method according to Reed and Muench (1938), as previously reported [60,61,62]. Briefly, 10-fold dilutions of the cell lysates in complete DMEM were used to infect a confluent monolayer of MDBK cells with four replicates for each dilution. Serial dilutions of BCoV were added to MDBK cells cultivated in 96-well plates, and 120 h after infection, the cells were monitored for the CPE, using an inverted optical microscope, considering that in every plate, four wells were used as a virus-free control. The titer was then calculated according to the well number with the CPE, after growing the cells at 37 °C for 120 h, according to the Reed–Muench method [62].

Moreover, the CPE was evaluated at 120 h p.i., after fixing the cells with methanol and staining them with crystal violet (0.1% *w/v*) (Sigma-Aldrich, St. Louis, MI, USA) [36].

### 4.7. Gene Expression

To assess the expression levels of the gene encoding the viral NP, MDBK cells, treated or not with SphA and SphB, were infected with BCoV (MOI = 0.5) for 48 h. Subsequently, the total RNA was collected using TRIzol reagent (Thermo Fisher, Waltham, MA, USA) and reverse-transcribed to cDNA using an all-in-one RT MasterMix (Applied Biological Materials, Richmond, BC, Canada). Real-time PCR was conducted by amplifying 2 μL of cDNA with BlasTaq 2 qPCR MasterMix (Applied Biological Materials, Richmond, BC, Canada) and 0.1 μM primers obtained from Eurofins. The sequences of the primers used for glyceraldehyde 3-phosphate dehydrogenase (GAPDH) were forward (5′-CGGAGTCAACATTTGGTCGTAT-3′) and reverse (5′-AGCTTCTCCATGGTGGGGGGTGGTGAAGAC-3′); for NP, forward (5′-GGACCCAAGTAGCGATGAG-3′) and reverse (5′-GACCTTCCTGAGCCTTCAATA-3′) [63]. The Relative Ct (the threshold cycle) of the gene of interest was normalized to the housekeeping gene (GAPDH). Finally, the mRNA levels were calculated using the 2^−ΔΔCt^ method.

### 4.8. LysoRed Staining

BCoV-infected cells at an MOI of 0.05, treated with SphA (2.5 μM) and SphB (1 μM), and incubated for 72 h were stained with CytoPainter LysoRed indicator reagent (Abcam Cambridge, UK) and incubated following the user manual. After that, the cells were washed and analyzed using a microscopic ZOE Fluorescent Cell Imager (Bio-Rad Laboratories, Hercules, CA, USA) [64].

### 4.9. Statistical Analysis

The results were expressed as means ± S.D.s. One-way ANOVAs with Tukey’s post hoc test and Student’s t-test were assessed using GraphPad Prism, version 10.0 (GraphPad Software Inc., San Diego, CA, USA); *p* < 0.05 was statistically significant.

### 4.10. Computational Studies of bAhR Interactions with SphA and SphB

The three-dimensional model of the bovine AhR (bAhR) (residues from Met^1^ to Lys^400^) was built, as described in a previous publication [36], on the basis of the primary sequence, using the AlphaFold 3.0 methodology with default parameters [65]. The stereochemical quality of the selected representative structural model of the bAhR was evaluated by analyzing the Ramachandran plot generated using the software PROCHECK v. 3.5 [66]. Successively, the software AutoDock 4.0 was used to dock the two ligands, SphA and SphB, to the bAhR^1−400^ structural model. In particular, the molecular docking protocol included the following steps: (1) The preparation of the input coordinate files for the ligands and the receptor, using AutoDockTools. The starting structures were prepared in order to include all the information needed for the docking protocol, such as polar hydrogen atoms, spatial charges, atom types, and torsional degrees of freedom. For SphA and SphB, the three-dimensional structure was built using the 3D tool of the ChemDraw 19.0 program, whereas the bAhR structure was prepared by adding missing hydrogen atoms and assigning appropriate Gasteiger charges to the reference AlphaFold structural model. (2) The pre-calculation of the grid maps, using the AutoGrid tool. The grid map is a 3D lattice of regularly spaced points, entirely or partially surrounding and centered in a specific region of the bAhR receptor. In our case, we fixed the grid box of a size of 40 × 40 × 40 points with a spacing of 0.375 Å in the middle of the bAhR TAD domain responsible for ligand binding. (3) The calculation of the docking structural models, using the AutoDock routine. The file containing the parameters required for the docking protocol was created using AutoDockTools. In particular, we used the Lamarckian Genetic Algorithm (LGA) as the searching method. Minimized ligands were randomly located within the grid box, and the docking protocol started with a quaternion and torsional steps of 3 and 4 torsional degrees of freedom for SphA and SphB, respectively. The number of energy evaluations was 2,500,000, and the run number was 100. After the docking simulation, the structures of each complex for both ligands were clustered using a backbone RMSD cutoff threshold of 2 Å. The most stable binding mode was selected from the cluster with the lowest binding free-energy, indicating a higher binding affinity. Moreover, each of the selected representative three-dimensional models was further validated by evaluating its structural stability through the calculation of the binding energy, using PRODIGY-LIG v. 1.1.0 software, which was able to produce results comparable with those obtained experimentally [67,68]. All the structures were analyzed and visualized using PyMoL [69] and Chimera [70].

## Data Availability

The data that support the findings of this study are available from the corresponding authors upon reasonable request.

## Supplementary Materials

The supporting information can be downloaded at https://www.mdpi.com/article/10.3390/ijms26157045/s1:
