# Supplementary material for: Structural Features and In Vitro Antiviral Activities of Fungal Metabolites Sphaeropsidins A and B Against Bovine Coronavirus
Source: Int J Mol Sci. 2025 Jul 22;26(15):7045. doi: 10.3390/ijms26157045 (PMC12346457; doi:10.3390/ijms26157045)
Supplement: Supplementary file 1 [file ijms-26-07045-s001.zip › ijms-3676037-supplementary.pdf]

Supplementary Materials

Structural Features and In Vitro Antiviral Activities of Fungal Metabolites Sphaeropsidins A and B Against Bovine Coronavirus

Luca Del Sorbo [1,†], Maria Michela Salvatore [1], Clementina Acconcia [2,†], Rosa Giugliano [1], Giovanna Fusco [3], Massimiliano Galdiero [4], Violetta Iris Vasinioti [5], Maria Stella Lucente [5], Paolo Capozza [5], Annamaria Pratelli [5], Luigi Russo [2], Rosa Iacovino [2,*], Anna Andolfi [6,*] and Filomena Fiorito [1]

[1] Department of Veterinary Medicine and Animal Production, University of Naples Federico II, 80137 Naples, Italy; luca.delsorbo2@studenti.unina.it (L.D.S.); mariamichela.salvatore@unina.it (M.M.S.); rosa.giugliano@unicampania.it (R.G.); filomena.fiorito@unina.it (F.F.)
[2] Department of Environmental, Biological and Pharmaceutical Sciences and Technologies, University of Campania Luigi Vanvitelli, 81100 Caserta, Italy; clementina.acconcia@unicampania.it (C.A.); luigi.russo2@unicampania.it (L.R.)
[3] Istituto Zooprofilattico Sperimentale del Mezzogiorno, 80055 Portici, Italy; giovanna.fusco@izsmportici.it
[4] Department of Experimental Medicine, University of Campania Luigi Vanvitelli, 80138 Naples, Italy; massimiliano.galdiero@unicampania.it
[5] Department of Veterinary Medicine, University of Bari, 70010 Valenzano, Italy; violetta.vasinioti@uniba.it (V.I.V.); mariastella.lucente@uniba.it (M.S.L.); paolo.capozza@uniba.it (P.C.); annamaria.pratelli@uniba.it (A.P.)
[6] Department of Chemical Science, University of Naples Federico II, 80126 Naples, Italy
* Correspondence: rosa.iacovino@unicampania.it (R.I.), andolfi@unina.it (A.A.)
† These authors contributed equally to this work.

Academic Editors: James K. Bashkin and Makoto Kimura

Received: 16 May 2025
Revised: 25 June 2025
Accepted: 18 July 2025
Published: date

Citation: Del Sorbo, L.; Salvatore, M.M.; Acconcia, C.; Giugliano, R.; Fusco, G.; Galdiero, M.; Vasinioti, V.I.; Lucente, M.S.; Capozza, P.; Pratelli, A.; et al. Structural Features and In Vitro Antiviral Activities of Fungal Metabolites Sphaeropsidins A and B Against Bovine Coronavirus. *Int. J. Mol. Sci.* **2025**, *26*, x. https://doi.org/10.3390/xxxxx

Copyright: © 2025 by the authors. Submitted for possible open access publication under the terms and conditions of the Creative Commons Attribution (CC BY) license (https://creativecommons.org/licenses/by/4.0/).

Supporting information list

Figure S1. (A) Schematic representation of the domain organization of the bovine Aryl Hydrocarbon Receptor (bAhR). These domains are highlighted along the sequence from residues 1 to 400. (B) Three-dimensional structural model of bAhR (residues 1–400) predicted using AlphaFold 3.0.The regions corresponding to the basic helix-loop-helix (bHLH) domain (green), involved in DNA binding and dimerization, PAS-A domain (purple) and PAS-B domain (cyan), which plays a role in transcriptional activation, and transactivation domain (TAD) (blue), which plays a role in transcriptional activation. The uncolored regions correspond to disordered segments. This structural model was used for molecular docking studies to investigate ligand-receptor interactions.
Figure S2. ^{1}H NMR spectrum of Sphaeropsidin A (Sph A) ($CDCl_3$, 400 MHz).
Figure S3. ^{1}H NMR spectrum of Sphaeropsidin B (Sph B) ($CDCl_3$, 400 MHz).
Table S1. Representative table of the residues of the bAhR receptor involved in interactions with SphA, as determined by docking analysis. The table illustrates the specific residues and their corresponding interactions, including hydrogen bond and hydrophobic contacts.
Table S2. Representative table of the residues of the bAhR receptor involved in interactions with SphB, as determined by docking analysis. The table illustrates

the specific residues and their corresponding interactions, showing that hydrophobic contacts exclusively stabilize the interaction.

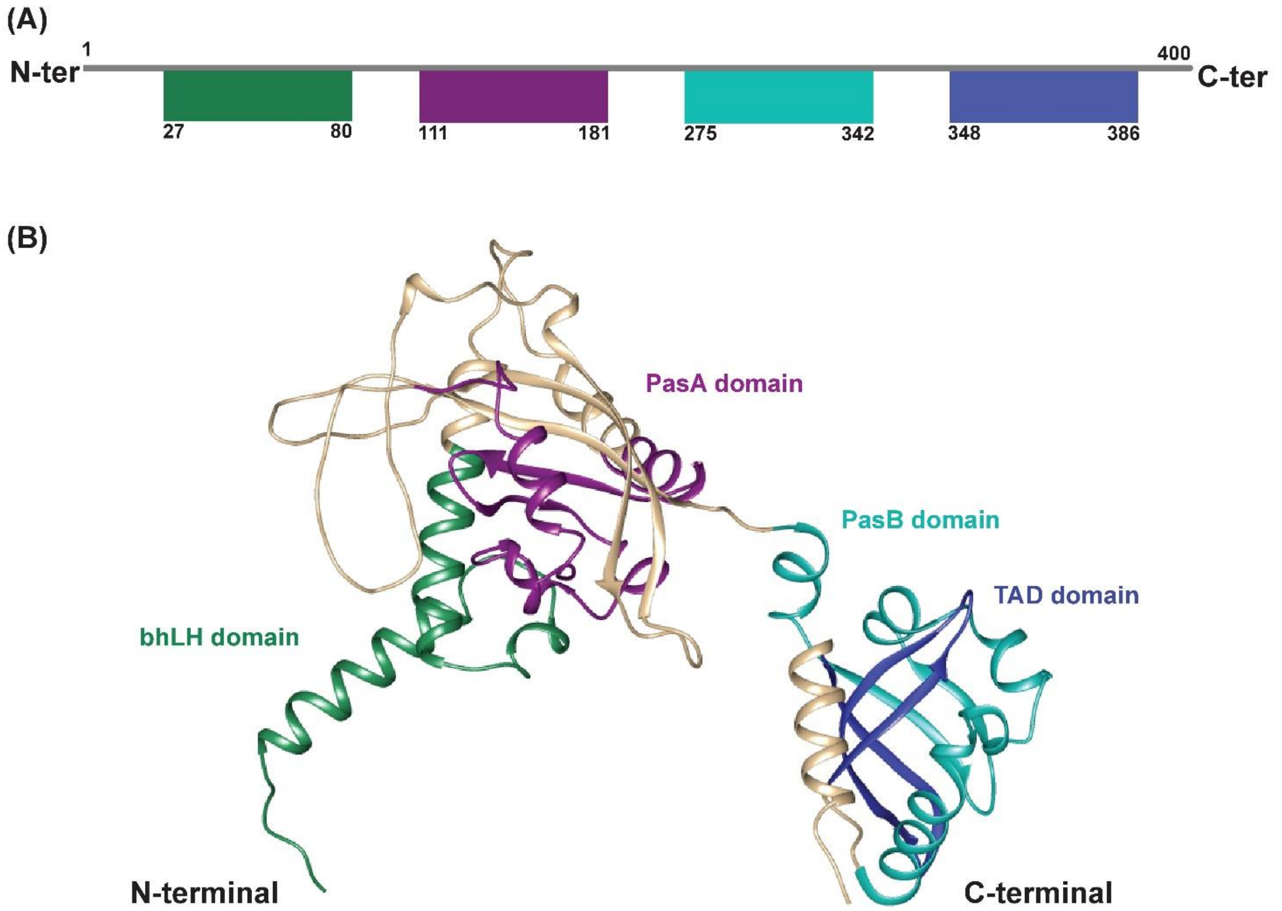

Figure S1. (A) Schematic representation of the domain organization of the bovine Aryl Hydrocarbon Receptor (bAhR). These domains are highlighted along the sequence from residues 1 to 400. (B) Three-dimensional structural model of bAhR (residues 1–400) predicted using AlphaFold 3.0.The regions corresponding to the basic helix-loop-helix (bHLH) domain (green), involved in DNA binding and dimerization, PAS-A domain (purple) and PAS-B domain (cyan), which plays a role in transcriptional activation, and transactivation domain (TAD) (blue), which plays a role in transcriptional activation. The uncolored regions correspond to disordered segments. This structural model was used for molecular docking studies to investigate ligand-receptor interactions.

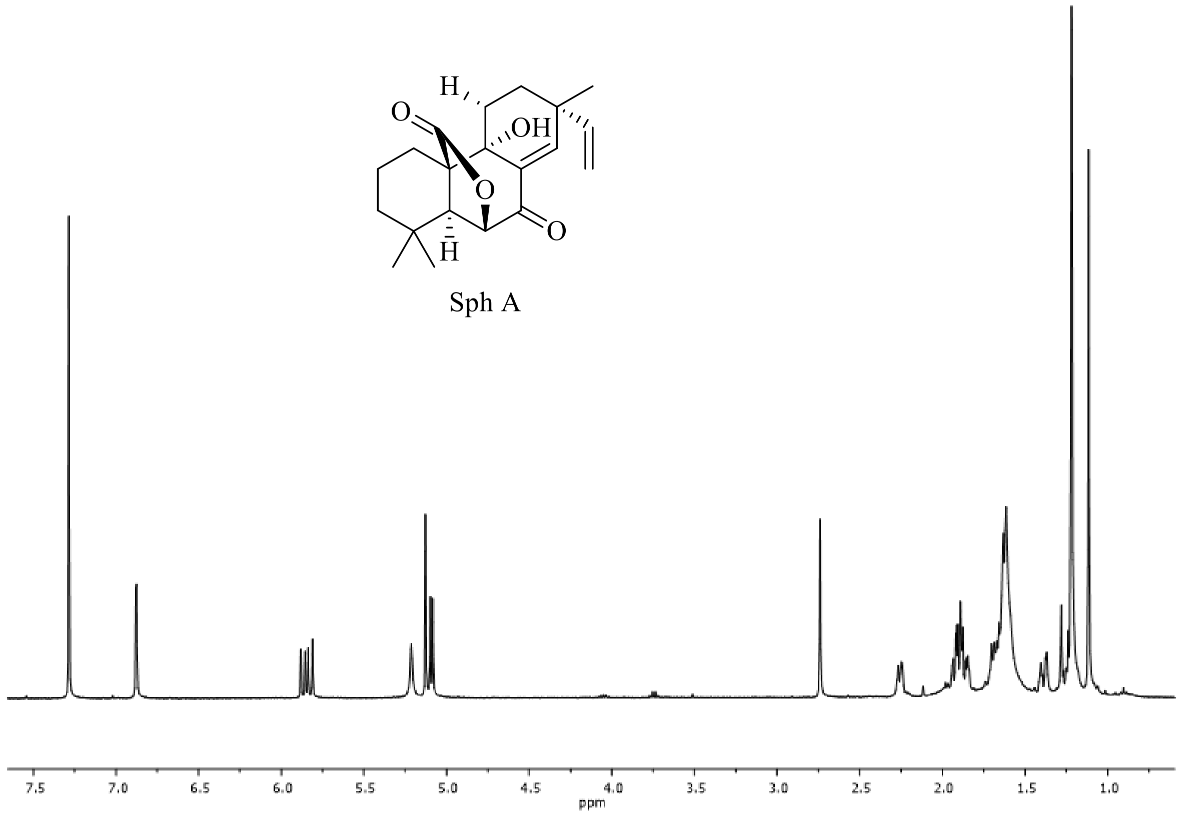

Figure S2. ^{1}H NMR spectrum of Sphaeropsidin A (Sph A) ($CDCl_3$, 400 MHz).

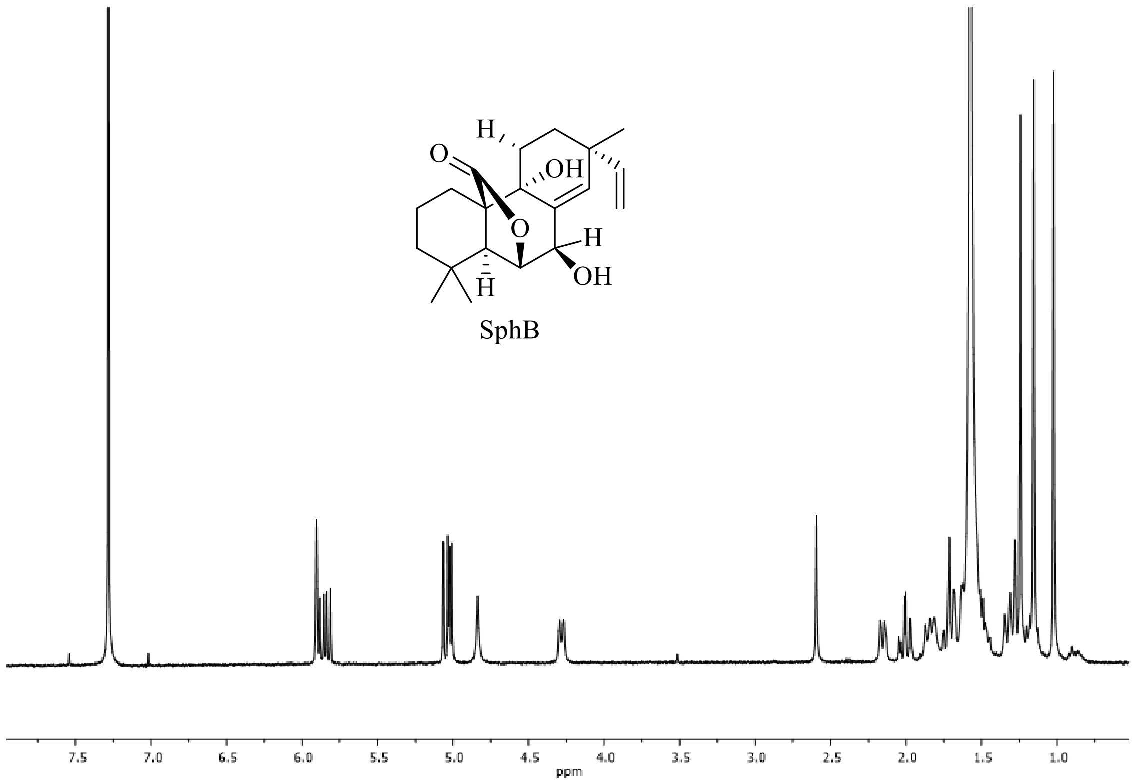

Figure S3. ^{1}H NMR spectrum of Sphaeropsidin B (Sph B) ($CDCl_3$, 400 MHz).

Table S1. Representative table of the residues of the bAhR receptor involved in interactions with SphA, as determined by docking analysis. The table illustrates the specific residues and their corresponding interactions, including hydrogen bond and hydrophobic contacts.

**Index**	**Hydrophobic Interactions**	**Hydrogen Bonds**
1	Phe 294	Ser 364
2	Val 324	
3	Tyr 335	
4	His 336	
5	Ile 348	
6	Phe 350	
7	Leu 352	
8	Ala 366	

Table S2. Representative table of the residues of the bAhR receptor involved in interactions with SphB, as determined by docking analysis. The table illustrates the specific residues and their corresponding interactions, showing that hydrophobic contacts exclusively stabilize the interaction.

**Index**	**Hydrophobic Interactions**
1	Phe 294
2	Tyr 321
3	Val 324
4	Met 329
5	His 336
6	Ile 348
7	Phe 350
8	Ala 366
